# A Pilot Study on the Knowledge, Attitude, and Practice of Mothers About Their Children’s Vaccination in a Medical Institute in Jharkhand, India

**DOI:** 10.7759/cureus.61478

**Published:** 2024-06-01

**Authors:** Partha K Chaudhuri, Abha Madhur, Pratik Sarkar, Kamal Narayan Prasad, Jyotsna Singh

**Affiliations:** 1 Department of Paediatrics, Rajendra Institute of Medical Sciences, Ranchi, IND; 2 Department of Paediatrics, Government Dental College and Hospital, Rahui, IND

**Keywords:** practice, knowledge, mother, children, vaccine-preventable diseases, vaccination

## Abstract

Introduction

Immunisation is one of the key public health instruments to combat childhood morbidity and mortality. However, the lack of mothers' knowledge and motivation to vaccinate their children has affected vaccination programs and vaccination coverage rate in the state of Jharkhand. Therefore, addressing this knowledge gap, our study aims to evaluate the extent of mothers’ understanding of the effects and aspects of vaccination for their children.

Materials and method

This is a cross-sectional study conducted at the paediatric vaccination clinic of Rajendra Institute of Medical Sciences (RIMS), Ranchi between October 2022 and September 2023. The sample population included 200 mothers as participants (18 years and above). The survey was done with a self-administered questionnaire of questions about socio-demographic factors, mothers’ knowledge, and mothers' practices, and answers were consolidated in the form of a table.

Results

The majority of participants in this study were below 25 years of age and were literate. The missed vaccination percentage was also significantly higher among illiterates, mothers below 30 years of age, and unemployed ones. Among the respondents, 73.3% of illiterate mothers, 56% of those below 30 years of age, and 64% of unemployed mothers missed their children’s vaccination schedule. Among the mothers, 75% did not know the names of vaccine-preventable diseases. Of the respondents, 50% believed intercurrent illnesses like fever and the common cold to be side effects and contraindications of vaccines. Among the mothers, 65% never posed any questions to the paediatrician. Of the mothers, 97% safely kept the vaccination card and 82% relied on government or public health centres for vaccination purposes.

Conclusion

The majority of our population was in favour of vaccinating their children but there existed a huge lacuna in their knowledge about vaccination. This study concludes that firmer measures have to be exercised to bridge this knowledge gap. Only this can improve the vaccination coverage rate.

## Introduction

Vaccines have played a heroic role in public health success stories as witnessed during the COVID-19 period. These have undeniably contributed to protection from communicable diseases and associated mortalities. Immunising children against vaccine-preventable diseases (VPDs) can greatly reduce childhood morbidity and mortality. As per the WHO factsheet on immunisation coverage, with >85% vaccine coverage, most countries of the world have been able to contain vaccine-avoidable diseases to a large extent, and the number of children missing out on any vaccination, i.e., zero-dose children improved from 18.1 million in 2021 to 14.3 million in 2022. With ~ 5 lakh (0.5 million), under-five deaths caused either directly or indirectly by VPDs, the figures for India do leave room for further improvement [1,2]. The Mother and Child Protection Card (MCP Card) is issued for the continuum of care through the Integrated Child Development Services (ICDS) scheme of the Ministry of Women and Child Development and the National Rural Health Mission (NRHM) of the Ministry of Health and Family Welfare, Government of India, serves as a tool for monitoring and providing complete immunisation to infants and children. Information on vaccination coverage collected and collated from the child’s health card and direct reporting from the mother is one of the key aspects. The figures from the National Family Health Survey 2019-2021 (NFHS 5), Ministry of Health and Family Welfare, Government of India show that only 54.9% of children below six years and 74% of those between 12-24 years of age, received immunisation in Jharkhand state of India [3]. Ignorance and lack of motivation in mothers is one of the prime causes behind this. Unvaccinated individuals pose public health risks to communities because of their vulnerability to spreading communicable diseases.

The Strategic Advisory Group of Experts (SAGE) has identified the factors associated with disinclination towards vaccination: hesitancy towards childhood and adolescent immunisations, parental vaccine refusal, complacency, lack of confidence, and convenience [4]. Although no single method is sufficient to check this attitude, counselling and communication are effective to a large extent [5]. The Universal Immunisation Programme of India covers VPDs on a large scale; however, the reluctance of vaccine-hesitant families leads to outbreaks [6]. In such a scenario, the maternal awareness of the importance of vaccination and the knowledge thereof, extends beyond the individual well-being, to the realm of community immunity and hence the broader spectrum of the public health landscape [7].

Since the mother is an integral part of the post-natal care and is the direct caregiver of the child right from infancy, hence study has involved mothers only. In our society, the father is generally away at work while the mother stays with the baby. In the case of working mothers also, they generally stay full-time with the baby during maternity leave within which the immunisation schedule is normally initiated. Also, mothers always accompany the child during the immunisation process and are conscious of the side effects, if these show up in the child. Hence our study focuses on mothers’ knowledge of vaccination and explores the relationship between the mother and the pediatrician considering the aspects of age, literacy level, and occupation. This study is also aimed at understanding the reasons behind non-compliance, mothers’ practice and attitude towards immunising their child, or the failure to immunise eligible children as per the vaccination schedule where surprisingly, needle fear on the part of the child also plays a part [8]. This study may help identify the factors that influence vaccination rates and thus improve the coverage rates. To this end, we conducted a questionnaire-based exercise for all the participant mothers who brought their children to the paediatric vaccination clinic at Rajendra Institute of Medical Sciences, Ranchi, Jharkhand, India.

## Materials and methods

Study design and setting

This is a cross-sectional study done in the paediatric vaccination clinic at Rajendra Institute of Medical Sciences (RIMS), Ranchi, by convenience sampling, during the period of October 2022 to September 2023. The inclusion criterion was mothers over 18 years who visited the clinic for immunisation of their children. The mothers who were above 18 years of age were excluded from the study. The study was done after obtaining the proper informed consent from the participants and an ethical clearance which was approved and passed by the Institutional Ethics Committee of RIMS, Ranchi. The approval number was IEC/R/11/24.

The methodology included a face-to-face in-person interview. For this purpose, a self-administered, pre-tested, structured questionnaire focussing on the mother’s attitude, practice, and knowledge about vaccination was framed. The interviewer also noted the mothers’ socio-demographic details including age, employment status, and educational status. During communication, information about vaccination specifics, knowledge about vaccines, safe-keeping of vaccination cards, preference of vaccination venue, delay of vaccination, and its erstwhile reasons were also procured.

Data encompassed socio-demographic details and characteristics (i.e., age, employment status, educational status), and characteristics about the children (i.e., number of children, age, and gender). We also delved into vaccination specifics, sources of information, communication with the paediatrician, delay of vaccination and its reasons, and vaccine knowledge.

The job of the mothers was broadly categorised into employed, and housewives. Education level was stratified into four groups, namely, illiterates, <10th standard, 10th pass (matriculation), and higher secondary passed (10+2 standard). To ascertain the main sources of information on vaccination knowledge, participants were asked, "What is your main source of information about vaccinating your children?" Response options included paediatricians and nurses. To understand the reasons for potential delays in vaccination, participants were asked, "Primary reasons behind postponing your child’s vaccination?” Responses included common cold, fever, diarrhoea, cost issues or travelling concerns. This study involved a total of 200 mothers as participants who responded to the interview.

Data processing and statistical analyses

The data was collected and collated on Microsoft Office Excel (Microsoft Corporation, Redmond, WA) and analysed by Software for Statistics and Data Sciences (STATA), version 18 (StataCorp LLC, College Station, TX, USA). Descriptive statistics were used to summarise the data of the study participants and present them as percentages. Regression analyses were done to identify associations between knowledge of VPDs, missed vaccination and socio-demographic data. Statistical significance was considered with p-value<0.05.

## Results

This study involved a total of 200 mothers as participants who responded to the interview.

Table 1 presents the age distribution, educational, and employment status of the participating mothers. Our analysis shows that the majority of the participants were either 25 years of age or below. This age group comprised 41% of the participants followed by mothers within the 31-35 years age group (38%). Among the mothers 15% were illiterate, 38% were non-matriculates followed by 35.5% matriculates (10th standard pass outs) and high school qualifiers (11.5%). Of the mothers, 81.5%, consisting of 163 participants were housewives or homemakers as against 18.5% of the employed ones.

**Table 1:** Characteristics of study mothers (n = 200)

Socio-demographic characteristics	n (%)
Mother’s age	
≤25	82 (41)
26-30	32 (16)
31-35	38 (19)
36-40	26 (13)
≥41	22 (11)
Mother’s education	
Illiterate	30 (15)
< Matriculation (10^th^)	76 (38)
10^th^ pass	71 (35.5)
Intermediate (10+2) pass	23 (11.5)
Mother’s occupation	
Housewife/homemaker	163 (81.5)
Employed	37 (18.5)

The data from our study portrayed in Table 2 shows that out of 170 literate participants, 50 of them possessed knowledge of VPDs whereas amongst the rest 30 participants, not even a single one possessed any knowledge about the same. Our study highlighted the finding that literate and educationally qualified mothers knew more about VPDs than others. Although 29.4% of literate mothers knew about VPDs, 30.5% of them missed their children’s vaccine shots whereas a whopping 73.3% of illiterate participants have made the same gaffe as shown in Table 2. Also, mothers of age >30 years showed a better perception of VPDs and similarly employed mothers had a better knowledge of the same. Among the mothers, 54.6% aged >30 years (n=47) showed a better perception of VPDs and had a lower missed vaccination percentage amounting to 20%. Interestingly, 49.1% (n=56) of mothers below 30 years of age reflected a careless attitude and missed vaccination. Employment status also mirrored similar findings by demonstrating that huge numbers (94.6%) of employed mothers knew about VPDs, as against 9.2% for unemployed mothers. The results of the statistical analysis conducted on mothers who were aware of VPDs revealed a statistically significant positive correlation with age, educational and socioeconomic status with a p-value of 0.013.

**Table 2:** Relationship of socio-economic and demographic characteristics of mothers and their knowledge and practice regarding vaccination of their children

	Knowledge of vaccine-preventable diseases n (%)	Missed vaccination percentage n (%)
Education level		
Literate (170)	50 (29.4%)	52 (30.5%)
Illiterate (30)	0	22 (73.3%)
Age		
<30 years (114)	3 (2.6%)	56 (49.1%)
>30 years (86)	47 (54.6%)	18 (20%)
Socio-economic status		
Employed (37)	35 (94.6%)	10 (26%)
Unemployed (163)	15 (9.2%)	64 (39%)
p-value	0.013	0.016

Among the respondents, 73.3% of illiterate mothers missed the vaccination schedule in contrast to 30.5% of their literate counterparts. Similarly, 56% of the mothers below 30 years and 64% of the unemployed mothers missed their children’s vaccination schedule. Table 2 also shows that employed mothers who had a better knowledge of VPDs got their missed vaccination percentage to be significantly lower (26%) whereas in the case of unemployed mothers, it was 39%. Upon statistical analysis, we obtained a significant correlation (p-value= 0.016) between non-adherence to the vaccination schedule with age, educational status (specifically, illiteracy), and employment status of the mothers.

Table 3 provides a summary of the results about mothers' knowledge of vaccination. A substantial majority of the mothers interviewed (75%) did not know the names of the diseases against which vaccines are given, while 25% did have the information. Among the respondents, 78% (n=156) of mothers mentioned the vaccines’ side effects, and the remainder 22% believed that vaccines have no side effects. Of the respondents, 50% mentioned fever as the primary side effect followed by fever with pain (22%), pain and swelling (4%), and rash (4%). About 70% (n=140) mentioned that intercurrent illness of fever, common cold, or loose stool would require deferring the immunisation. The remainder 30% believed vaccines ought to be received despite those conditions. Half of the participants mentioned common cold and fever as the major contraindication of vaccination, and 20% believed it to be diarrhoea. Of the respondents, 30% did not visualise any contraindication associated with vaccines.

**Table 3:** Summary of mothers’ knowledge about disease name, side effects and contraindications of vaccines

Mothers’ knowledge	n (%)
Diseases’ name	
Know	50 (25)
Did not know	150 (75)
Vaccines’ side effects	
Fever	100 (50)
Rash	8 (4)
Pain and swelling	8 (4)
Fever and pain	40 (20)
No side effect	44 (22)
Vaccines’ contraindication	
Common cold, fever	100 (50)
Diarrhoea	40 (20)
No contraindication	60 (30)

Table 4 summarises mothers’ practices. Approximately two-thirds of the mothers’ population (65%) stated that they did not pose any questions to their child's physician concerning vaccination. In contrast, one-third (35%) reported making inquiries when they visited their children's physician. Regarding the query about the primary sources of immunisation information, a majority, 65% (n=130) of respondents, identified either the delivery room or health clinic nurse as a source to guide them. Most mothers (82%) preferred government services for immunisation, 8% of mothers preferred private office and to the rest 10%, the place of vaccination did not matter. Most of the mothers knew the importance of keeping the vaccination card safe and agreed that they should not lose their children’s immunisation cards. Mothers generally, 97% (n=194) believed that the vaccination card is an important document to be kept safely as vaccination protects their child from diseases. Only 3% of mothers lost their vaccination cards.

**Table 4:** Summary of mothers’ practice

Mothers’ practice	n (%)
Ask question from doctors	70 (35)
Ask question from delivery room nurses	130 (65)
Save vaccination card	
Keep safely	194 (97)
Lose card	6 (3)
Preference of vaccination place	
Govvernment/public health centre	164 (82)
Private office	16 (8)
No difference	20 (10)

## Discussion

In India, under the Universal Immunisation Programme (UIP) and Extended Programme of Immunisation (EPI), vaccines are administered to infants and children to protect them from diphtheria, pertussis, tetanus, polio, measles, rubella, severe forms of childhood tuberculosis, hepatitis B, meningitis, and pneumonia caused by Hemophilus influenzae type B. The India-EPI Factsheet 2023, released by WHO, reflects a positive scenario of >80% vaccine coverage with a negative shade by citing >10% drop-out rate for DTP-HibHepB1 to DTP-Hib-HepB3 vaccine and 1,125,995 zero-dose children who have not been covered by the immunisation programme [9]. To bring such children under vaccine coverage, it is very essential to understand and analyse the knowledge, attitude, and practice of mothers regarding vaccination. With this objective in mind, this study was performed at RIMS, Ranchi in the state of Jharkhand which is still developing in terms of public health in India.

A mother’s education is one of the most decisive factors in her child's health outcomes. If she has limited knowledge and understanding of the vaccination schedule and dosages, she is less likely to participate and complete her child's immunisation as per the schedule [10]. Among the study population, 73.3% were illiterate. Education empowers women with a positive attitude towards vaccination. A Saudi Arabian study by Alghofaili et al., pointed to the determinants like illness of the child on the day of vaccination, parental education, parental careless attitude, and vaccine beliefs for the vaccination delays [11]. In another study by a group in China, a positive association was drawn between parental education and hesitance for their children’s vaccination [12]. Our study also concluded the positive effect of a mother’s educational status on her child’s immunisation. Maternal education is one of the determining factors of childhood immunisation and is hence important for the physical well-being of the family [10]. Educated mothers are more aware of the schedule, dosages and advantages of childhood immunisation.

Vaccination delay or missed shots impacts the child’s health and overall well-being. It might lead to dangerous diseases leading to serious complications. Alongside this, the insincere attitude towards vaccination leads to compromised herd immunity in society as reflected in the study by Banjari et al. [13]. In our study, most of the mothers expressed a positive attitude toward the vaccination of their children. We also found a statistically significant correlation between literacy, age, and employment with awareness of VPDs (p-value=0.013) and disrupted vaccination schedules (p-value=0.016). Similar findings have echoed in a previous study by Wagner et al., from both developed as well as developing countries [8]. Also, most mothers obtained information about their child’s vaccination from nursing staff or medical social workers rather than with the pediatrician during appointments which again reflects their positive attitude. However, notably, respondents lacked awareness of VPDs and their associated vaccines. Of the illiterate mothers, not a single mother knew about VPDs whereas 69.4% of literate mothers knew. This trend seems to be in line with a study in Canada where mothers had limited knowledge about VPDs despite their intention to get their infants vaccinated [14]. Sometimes, vaccination deferral arises due to the disproportionate importance given to minor intercurrent diseases. In a study conducted in Delhi, the reasons for the inconsistent follow-up and immunisation delay were mostly due to a lack of faith in the effectiveness of vaccines, lack of knowledge about booster doses, and very surprisingly, considering oral polio vaccine to be the only important vaccine [15]. Our study showed that 75% of the mothers were ignorant about the names of VPDs. Hence maternal attitude in such cases remains to be changed.

In the present study, a large percentage (82%) of our study population preferred government and public health centres for vaccination, even if these had inadequate communication facilities. Mothers trusted these centres because they provided better quality vaccines free of charge. Another reason is that nurses who provide information and advice to mothers, thoroughly impact maternal decisions and also provide reassurance to mothers regarding the safety of vaccines. Our findings align with the outcomes of preceding studies by van Erp [16]. Hence socio-economic factors and mothers’ education play an important role in this aspect. Lower economic status is generally linked to under-vaccination rates whereas better economic status leads to vaccination acceptance and adherence to the schedules. Hence financial constraint is also a determinant of vaccine acceptance.

In a study by Balgovind and Mohammadnezhad in Fiji about the perceptions of healthcare workers towards childhood immunisation services, it was observed that an immunisation plan in the form of a vaccination card ensures timely immunisation and minimises forgetfulness and missed shots [17]. Literate mothers can follow the instructions on these cards. These cards also document immunisation status. Of the mothers, 97% who participated in this study ensured safe keeping of these cards which reflects their positive attitude.

Nakatudde et al. in 2019 in a tertiary hospital in Uganda observed that delaying vaccination can disrupt the intended purpose of the vaccination schedule, resulting in vaccine outcomes that may have adverse effects on herd immunity, morbidity, and mortality rates in children [18]. Hence this study holds implications for improving the public health initiatives accordingly. Our study findings underscore the significance of targeted efforts not only to maintain but also to enhance vaccination rates, particularly by addressing concerns related to maternal knowledge, attitude, and practice related to vaccine safety. This includes tailoring interventions for those with behaviours and attitudes indicative of such concerns, and also for individuals facing socioeconomic risk factors like lower educational levels and income. The most prominent determinant of instilling confidence in mothers is the effectiveness, value, and safety of recommended childhood vaccines [19,20]. In a cross-sectional study about the factors contributing to the delayed vaccination among children in Riyadh, Alghofaili et al. found that the apprehensions regarding vaccine safety can only be warded off by fostering continuous open communication between mothers and healthcare workers at all levels [11]. This is also implicated from our study that this communication can play a significant role in emphasising the importance of timely vaccination and raising awareness about vaccine safety, contraindications, and side effects. Healthcare workers acknowledge their important role in the immunisation process as the source of direct information and positive motivation for the parents. The mothers may also be motivated to administer multiple vaccines at a single visit [10,21]. Good knowledge about vaccines and vaccination communication strategies are the key factors in changing the negative and neutral attitudes of mothers toward immunisation [22]. Predictably, here 100% of illiterate mothers had missed vaccine shots. Also, 94.6% of the employed mothers knew about VPDs. Small interactive group meetings on vaccination, during prenatal courses and births to reduce vaccine hesitation [23]. This may also be adopted as one of the communication strategies. Understanding the causes of maternal knowledge, attitude, and hesitancy holds the key to shaping the methods of vaccine messaging, besides framing immunisation policies, recommendations and guidelines. Maternal attitude can be a both barrier and an enabler [24]. Hobani and Alhalal observed that running health education programmes will instill confidence, a positive attitude and self-efficacy in mothers, to make healthy decisions [25]. They will follow their children’s immunisation schedule to ensure timely vaccination.

Of the mothers, 50% believed fever was the only side effect. Such misconceptions can be allayed during periodic visits. Communication of health care workers with mothers will inform them of the associated side effects like swelling at the injection site, pain, fever and, rash; remind them of the next visit, the due vaccine doses, and compensatory vaccine doses in case of failure. They are hence encouraged to engage in concerted discussions with mothers, especially those with limited health literacy, to effectively convey the message of the benefits of vaccines to mothers and influence their beliefs, intentions, and behaviour. They should strengthen their interpersonal and communication abilities to motivate and guide the mothers.

Our cross-sectional study design provides a snapshot in time. Thus, it may not always be possible to establish causality. The present study is based on the questionnaire presented to the mothers who visited the vaccination clinic. Hence such mothers are already more aware of their outlook than the population who never showed up and are thus ignorant. This bias can be mitigated by a rigorous and random door-to-door survey which is better than a questionnaire-based round, thus making it our limitation. The small sample size, convenience sampling, and non-inclusion of fathers are also limitations of this study and may limit the generalisability of the findings. Social desirability bias or recall bias may also play a part in biasedness in self-reported data. Moreover, the majority of the participating mothers in our study are literate and supposedly have some knowledge of childhood vaccination, making it a limitation in itself.

We have observed from this study that maternal literacy and education, age, and employment are linked with their knowledge of VPDs and missed vaccine shots. Effective communication with them is essential to bring the children within the immunisation fold and raise awareness about the actual side effects. The findings from this study can impact broader public health strategies and vaccination programmes.

## Conclusions

Despite a forward outlook towards vaccination, mothers’ knowledge and information about vaccine-preventable diseases (VPDs) and childhood vaccination is poor. Besides, there is a huge communication gap between them and their physician. This unique opportunity to educate mothers and enhance their performance and knowledge on childhood vaccination is anticipated to be used by doctors, nurses, or other healthcare professionals. Herd immunisation is very useful in mitigating VPDs but becomes a distant reality due to vaccine ignorance, hesitancy, and lack of communication, so it calls for immediate action. Thus, collaborative efforts are essential to address the complex challenges hindering optimal vaccination coverage globally. Also, cost allocation in a planned manner to expand communication about vaccination is essential.

Longitudinal data collected over an extended time frame could provide more insights into how mothers’ and parents’ knowledge and practices evolve. Future studies may also involve fathers’ attitudes. Furthermore, the changes in government policies to ensure pre-natal courses on VPDs, discussions and suggestions to the would-be mothers, the improvement of mothers’ literacy, communication of mothers with health care workers regarding immunisation, and interpersonal communication by electronic media will impart a positive impact. Exposing the mothers to the benefits of immunisation and drawbacks of missed vaccine shots will bring a positive attitude in the mothers.
